# Frequency and factors associated with falls in adults aged 55 years or more

**DOI:** 10.1590/S1518-8787.2017051005409

**Published:** 2017-04-18

**Authors:** Sabrina Canhada Ferrari Prato, Selma Maffei de Andrade, Marcos Aparecido Sarria Cabrera, Renata Maciulis Dip, Hellen Geremias dos Santos, Mara Solange Gomes Dellaroza, Arthur Eumann Mesas

**Affiliations:** I Secretaria Municipal de Saúde. Prefeitura do Município de Cambé. Cambé, PR, Brasil; II Departamento de Saúde Coletiva. Centro de Ciências da Saúde. Universidade Estadual de Londrina. Londrina, PR, Brasil; III Departamento de Clínica Médica. Centro de Ciências da Saúde. Universidade Estadual de Londrina. Londrina, PR, Brasil; IV Programa de Pós-Graduação em Epidemiologia. Faculdade de Saúde Pública. Universidade de São Paulo. São Paulo, SP, Brasil; V Departamento de Enfermagem. Centro de Ciências da Saúde. Universidade Estadual de Londrina. Londrina, PR, Brasil

**Keywords:** Middle Aged, Aged, Accidental Falls, Risk Factors, Health Surveys

## Abstract

**OBJECTIVE:**

The objective of this study is to analyze the frequency and factors associated with falls in adults aged 55 years or more.

**METHODS:**

This is a study inserted into another population-based study with representative sample of persons aged 40 years or more of the urban area in a medium-sized municipality of the State of Paraná, Brazil, in 2011. That study obtained demographic and socioeconomic data and characteristics related to life habits, health conditions, and functional capacity (n = 1,180). In 2012, we selected all persons aged 55 years or more (n = 501). We have estimated grip strength and the occurrence of a fall since the last interview in 80.6% of the adults. The crude and adjusted odds ratios (OR) have been calculated by logistic regression according to a hierarchical model.

**RESULTS:**

The rate of fall was 24.3%. After adjustments, we could observe higher chances of falls among women (OR = 3.10; 95%CI 1.79–5.38), among persons aged 65 years or more (OR = 2.39; 95%CI 1.45–3.95), with poor sleep quality (OR = 1.78; 95%CI 1.08–2.93), and with low grip strength (OR = 2.31; 95%CI 1.34–3.97).

**CONCLUSIONS:**

Poor sleep quality and low muscle strength can be indicators of increased risk of falls and need assessments and interventions aimed at preventing them.

## RESUMO

**OBJETIVO:**

Analisar a frequência e fatores associados à ocorrência de quedas em adultos de 55 anos ou mais.

**MÉTODOS:**

Estudo inserido em outro de base populacional com amostra representativa de pessoas com 40 anos ou mais da área urbana de município de médio porte do Paraná em 2011. Foram obtidos dados demográficos e socioeconômicos, características referentes aos hábitos de vida, às condições de saúde e à capacidade funcional (n = 1.180). Em 2012, selecionaram-se todas as pessoas com idade igual ou superior a 55 anos (n = 501). Foram estimadas a força de preensão palmar e a ocorrência de queda desde a última entrevista em 80,6% delas. Foram calculadas *odds ratio*s (OR) brutas e ajustadas por regressão logística segundo modelo hierarquizado.

**RESULTADOS:**

A frequência de queda foi de 24,3%. Após ajustes, observaram-se chances maiores de queda entre mulheres (OR = 3,10; IC95% 1,79–5,38), entre pessoas com idade igual ou superior a 65 anos (OR = 2,39; IC95% 1,45–3,95), com qualidade do sono ruim (OR = 1,78; IC95% 1,08–2,93) e com baixa força de preensão palmar (OR = 2,31; IC95% 1,34–3,97).

**CONCLUSÕES:**

Qualidade ruim do sono e a baixa força muscular podem ser indicadores de maior risco de quedas e merecem avaliações e intervenções visando à prevenção desse agravo.

## INTRODUCTION

The occurrence of falls is related to a complex interaction of risk factors aggravated with aging. A literature review on the subject^2^ points as main risk factors: being female, age, polypharmacy, use of psychotropic substances, previous history of falls, visual impairment, cognitive decline, and environmental factors, such as slippery floors and poorly positioned furniture and carpets. In addition, some conditions or chronic degenerative diseases are associated with higher incidence of falls, such as obesity^26^, hypertension^2^, diabetes^28^, neoplasms^24^, neuropsychiatric diseases^2^ ^,^ ^28^, and osteomuscular diseases^26^. On the other hand, the regular practice of physical activity stands out as a protective factor^20^.

Disability and serious injuries are among the main consequences of a fall. They imply high economic and social costs, especially when there is impairment of the independence of the individual and the need for specialized care at home or in long-term institutions^2^.

Although there are several studies on falls in the elderly in the community, we can observe a concentration of studies among elderly persons with morbidities, weakened, and institutionalized^5^. The *Diretrizes de Prevenção de Quedas em Idosos* ^12^ (Guidelines for Prevention of Falls in the Elderly – 2010) demonstrate the importance of early identification of older adults at risk. In this context, the objective of this study is to analyze the frequency and factors associated with falls in individuals aged 55 years or more. The knowledge on this profile can direct the efforts of primary care professionals in preventing falls and maintaining functional independence.

## METHODS

This study is part of a larger population-based study (*Doenças Cardiovasculares no Estado do Paraná: mortalidade, perfil de risco, terapia medicamentosa e complicações* – VIGICARDIO)^23^, with representative sample of residents aged 40 years or more in the urban area of Cambé, State of Paraná, Brazil. The municipality has a population of 96,735 inhabitants, according to the 2010 Census^a^. The calculation of the sample of the study VIGICARDIO considered a margin of error of 3.0%, 95% confidence interval, prevalence of outcome of 50.0%, and increase for losses and refusals of 25.0%, resulting in 1,322 persons to be interviewed. The eighty-six census tracts of the urban area of Cambé were selected and the number of persons to be interviewed in each tract was defined according to the proportional distribution of residents by gender and age group (for every five years). After drawing the initial block, we draw the corner of the block in which we would initiate the route, counterclockwise, with a sampling interval of 1.2, in order to ensure the representation of all census tracts. Details of the sampling calculation and the process of selection of interviewees can be found in another publication^23^.

The data were obtained using home interviews. We obtained information on life habits, health conditions, use of drugs (with the presence of inserts, prescriptions, or packages), activities of daily living (ADL), and instrumental activities of daily living (IADL), using the scales of Katz et al.^10^ and Lawton and Brody^13^, respectively, and also sleep quality using the Pittsburgh index (PSQI)^4^. Weight and height were verified using a portable electronic scale and an inextensible measuring tape, respectively. We obtained three blood pressure measurements and considered the arithmetic average of the second and third measurements to define hypertension (≥ 140 mmHg for systolic blood pressure and/or ≥ 90 mmHg for diastolic blood pressure and/or use of antihypertensive medication)^23^.

The VIGICARDIO data collection took place between February and June 2011, amounting to 1,180 participants. For logistical reasons, individuals aged 55 years or more were selected for this study, corresponding to 501 participants. Between April and August 2012, we conducted a second home interview. Among those participants aged 55 years or more in the first study, 404 (80.6%) were located in the second survey. The losses occurred because of change of address (n = 51), hospitalization (n = 3), death (n = 11), refusal (n = 12), or they could not be located after three attempts (n = 20). The average time elapsed between the two data surveys was fourteen months (minimum of eleven and maximum of eighteen months). There was no significant difference between interviewees and losses regarding age, race, marital status, educational level, economic class, or presence of chronic diseases. Only gender showed significant difference (p = 0.02), with greater loss of men.

The dependent variable corresponded to self-reported accidental fall after the first home interview. The question used was: “Since the last interview, have you fallen?”

The following variables were obtained in the first home interview:

- Demographic and socioeconomic characteristics: gender, age, marital status, race, education, and economic class.
- Characteristics related to life habits and health conditions: leisure-time physical activity, smoking, body mass index, use of psychotropic drugs, sleep quality, report of medical diagnosis for the following morbidities: congestive heart failure (CHF), diabetes mellitus (DM), depression, chronic obstructive pulmonary disease (COPD), neoplasm, and hypertension.
- Characteristics related to functional capacity: visual and/or hearing impairment (self-reported), difficulty to go up or down the stairs, and difficulty to perform activities of daily living (ADL)^10^ and instrumental activities of daily living (IADL)^13^.

In the second interview, we verified grip strength using a hydraulic hand dynamometer, model SAEHAN SH5001, following the standards recommended by the American Society of Hand Therapists^7^.

For the bivariate and multiple analyses, age was grouped into 55 to 64 years and 65 years or more, race was grouped into white or yellow and black/brown/indigenous, and marital status was grouped into with and without a partner.

Schooling was classified by years of study (zero to three, four to seven, and eight years or more). Economic class was assessed according to the criteria of the *Associação Brasileira de Empresas de Pesquisas* (ABEP – Brazilian Association of Research Companies)^b^, grouped into class A/B, C, and D/E.

The recommendations of Haskel et al.^8^ were followed for the assessment of leisure-time physical activity. We considered as practitioners of physical activity those who reported to perform moderate-intensity physical activity (e.g., walking in fast pace, dancing) at least for 30 minutes, five or more times a week, and/or vigorous-intensity physical activity (e.g., running, playing soccer) at least for 20 minutes, three or more times a week. The ones that did not fit these criteria were considered as non-practitioners.

Visual impairment was divided into two categories: individuals without impairment or those who had some visual disorder that did not harm them in carrying out the activities of daily life, and individuals with exacerbated visual impairment (who only saw the outline of objects and needed to be guided on activities of daily living) or those who did not see.

We considered as dependent the persons who reported difficulty in performing two or more activities of daily living or two or more instrumental activities of daily living^11^.

The information related to the continuous use of drugs was collected using supporting elements (inserts, packages, bottles, boxes, or prescriptions) and questions related to the indication, location of where the drug was bought, and use of the drug. We used the Anatomical Therapeutic Chemical classification (N03-N07)^c^ for the identification of psychotropic drugs.

Sleep habits in the 30 days leading up to the first home interview were assessed using the Pittsburgh sleep quality index (PSQI)^4^. Values greater than five points were considered indicative of poor quality of sleep.

Grip strength was analyzed according to percentiles, separately for men and women. Because there is no cutoff point validated in the literature to define low muscle strength, persons were classified into: ≤ 25th percentile (low muscle strength) or > 25th percentile.

A portable electronic scale, Plenna brand, was used to measure weight, with a precision of 100 grams. Height was measured using an inelastic and inextensible measuring tape fixed at a wall without baseboard or door. We calculated the body mass index (BMI) for classification of obesity, according to the criteria of the World Health Organization (BMI < 30 = no, ≥ 30 = yes)^21^.

Considering that the variable grip strength was obtained only in the second interview, we carried out a cross-sectional data analysis. The measure of association used was the odds ratio (OR) for the bivariate analysis, and the binary logistic regression was carried out by a conceptual hierarchical model, using the backward stepwise method (Likelihood Ratio).

Variables with p < 0.20 in the bivariate analysis were selected to compose the hierarchical conceptual model. The model was defined in three levels: distal (demographic and socioeconomic characteristics), intermediate (characteristics related to life habits and health conditions), and proximal (characteristics related to functional capacity), considering the temporal precedence of the factors for a fall (Figure). We decided to keep the economic class for adjustment in the distal level in the modeling, regardless of the statistical outcome.

**Figure:**
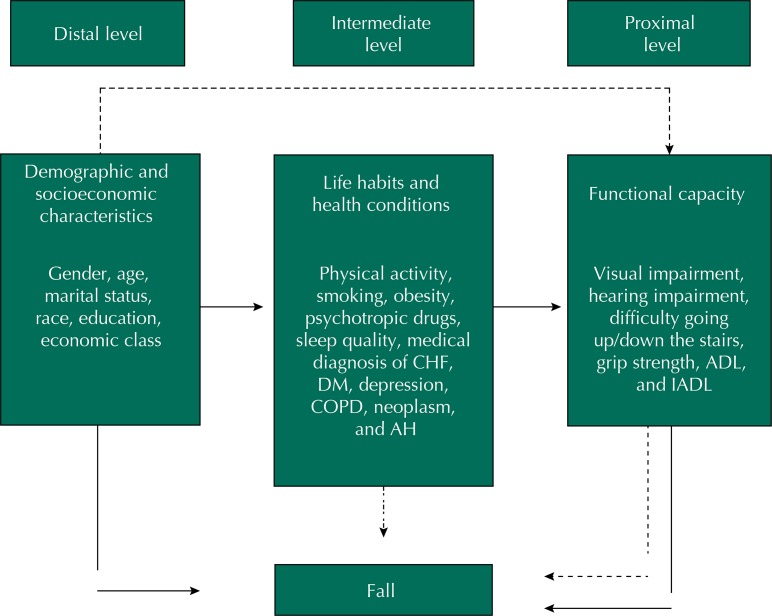
Hierarchical conceptual model of the factors associated with a fall in persons aged 55 years or more. Cambé, State of Paraná, Southern Brazil, 2011 to 2012.

BMI: body mass index; CHF: congestive heart failure; DM: diabetes mellitus; COPD: chronic obstructive pulmonary disease; AH: arterial hypertension; ADL: activities of daily living; IADL: instrumental activities of daily living

The variables of the distal level were adjusted by those of the same level. The variables of the intermediate level were adjusted by the variables with p < 0.10 in the previous adjustment and by variables of the same level, and the variables of the proximal level were adjusted by the variables with p < 0.10 in the previous adjustment and by variables of the same level. We excluded from the multiple analysis individuals with any field that was not informed or encoded as ignored. The level of significance adopted for the analyses was 5% (Wald test). For all variables, we established a reference category (OR = 1), considered as the lowest risk for a fall.

The statistical analyses were performed with the aid of the program SPSS 19.0.

This study was approved by the Research Ethics Committee of the *Universidade Estadual de Londrina* for the first (CAAE 0192.0.268.000-10) and second research (CAAE 0021.0.268.000-11). The participants signed the informed consent.

## RESULTS

The average age of the 404 individuals assessed in the two studies was 65.5 years (standard deviation of 7.1 years). More than half (59.4%) of the interviewees were female, 66.6% reported being white or yellow, 67.1% were married or had a stable union, and 54.2% were considered as economic class C. Average education was 4.4 years (standard deviation of 4.2 years). Of the total, 22.8% regularly practiced some physical activity and 14.6% were smokers. The continued use of at least one psychotropic drug was mentioned by 19.6% of the interviewees. The sleep quality of 39.4% of the population was considered poor, 20.0% reported medical diagnosis of depression, 16.1% of diabetes mellitus, 12.1% of chronic obstructive pulmonary disease, 6.7% of neoplasm, 40.1% of hypertension, and 4.5% of heart failure.

Just over one third (36.6%) of the interviewees mentioned difficulty going up or down the stairs, 12.4% had hearing impairment, and 10.9% had visual impairment. Most of the population under study was considered as independent to carry out activities (98.5%) and instrumental activities (85.9%) of daily living.

Fall from the first interview was mentioned by 24.3% of the individuals (95%CI 20.1–28.5) and, of this number, 64.3% (95%CI 54.8–73.8) reported a single fall. We observed an increase of falls according to an increase in age: 15.6% (95%CI 8.1–23.1) for individuals aged between 55 and 59 years, 19.0% (95%CI 11.9–26.1) for those aged between 60 and 64 years, and 31.3% (95%CI 24.8–37.8) for those aged 65 years or more.

In the bivariate analysis, we observed higher chances of falls (p < 0.05) among females, aged 65 years or more (Table 1), with poor sleep quality, diagnosis of depression (Table 2), difficulty going up or down the stairs, considered as dependents to carry out activities of daily living, and with low grip strength (Table 3).

**Table 1:** Risk factors (not adjusted) for a fall, related to the demographic and socioeconomic characteristics. Cambé, State of Paraná, Southern Brazil, 2011 to 2012. (N = 404)

Variable	Total	Fall (yes)		Not adjusted OR	95%CI	p^a^
	
	N	n	%			
Gender						
Male	164	24	14.6	1	-	
Female	240	74	30.8	2.60	1.56–4.34	< 0.001
Age (years)						
55 to 64	206	36	17.5	1	-	
≥ 65	198	62	31.3	2.15	1.35–3.44	0.001
Marital status						
With partner	271	63	23.2	1	-	
Without partner	133	35	26,3	1.18	0.73–1.90	0.449
Race						
Black/Brown/Indigenous	135	28	20.7	1	-	
White/Yellow	269	70	26.0	1.34	0.82–2.21	0.244
Education level (complete years)^b^						
0 to 3	88	22	25.0	1	-	
4 to 7	136	26	19.1	0.71	0.37–1.35	0.296
≥ 8	179	50	27.9	1.16	0.65–2.08	0.612
Economy class (ABEP)^b^						
A/B	119	26	21.8	1	-	
C	219	54	24.7	1.17	0.69–1.99	0.562
D/E	65	18	27.7	1.37	0.68–2.75	0.375

ABEP: *Associação Brasileira de Empresas de Pesquisa* (Brazilian Association of Research Companies).

^a^ Wald Chi-square.

^b^ Information missing for one interview.

**Table 2:** Risk factors (not adjusted) for a fall, related to life habits and health conditions. Cambé, State of Paraná, Southern Brazil, 2011 to 2012. (N = 404)

Variable	Total	Fall (Yes)		Not adjusted OR	95%CI	p^a^
	
	N	n	%			
Practice of physical activity						
Yes	75	18	24.0	1	-	
No	329	80	24.3	1.02	0.57–1.83	0.954
Smoking						
No	345	84	24.3	1	-	
Yes	59	14	23.7	0.97	0.51–1.85	0.918
Use of psychotropic substances^b^						
No	271	70	25.8	1	-	
Yes	132	28	21.2	0.77	0.47–1.27	0.310
Quality of sleep^c^						
Good	224	44	19.6	1	-	
Poor	157	49	31.2	1.86	1.16–2.98	0.011
Heart failure^d^						
No	386	96	23.8	1	-	
Yes	18	6	33.3	1.60	0.58–4.38	0.362
Diabetes mellitus^d^						
No	339	83	24.5	1	-	
Yes	65	15	23.1	0.93	0.49–1.73	0.809
Depression^d^						
No	323	69	21.4	1	-	
Yes	81	29	35.8	2.05	1.21–3.48	0.007
COPD^d^						
No	355	87	24.5	1	-	
Yes	49	11	22.4	0.89	0.44–1.82	0.753
Neoplasm^d^						
No	377	88	23.3	1	-	
Yes	27	10	37.0	1.93	0.85–4.37	0.114
Hypertension						
No	242	54	22.3	1	-	
Yes	162	44	27.2	1.30	0.82–2.06	0.265
Obesity^e^						
No	262	58	22.1	1	-	
Yes	133	38	28.6	1.41	0.87–2.27	0.159

COPD: Chronic obstructive pulmonary disease

^a^ Wald Chi-square.

^b^ Information missing for one interviewee.

^c^ Information missing for twenty-three interviewees.

^d^ Medical diagnosis reported by the interviewee.

^e^ Information missing for nine interviewees.

**Table 3:** Risk factors (not adjusted) for a fall, related to functional capacity. Cambé, State of Paraná, Southern Brazil, 2011 to 2012. (N = 404)

Variable	Total	Fall (Yes)		Not adjusted OR	95%CI	p*
	
	N	n	%			
Visual impairment						
No	360	89	24.7	1	-	
Yes	44	9	20.5	0.78	0.36–1.69	0.534
Hearing impairment						
No	354	81	22.9	1	-	
Yes	50	17	34.0	1.74	0.92–3.28	0.089
Difficulty of going up/down the stairs						
No	256	46	18.0	1	-	
Yes	148	52	35.1	2.47	1.55–3.94	< 0.001
ADL						
Independent	382	88	23.0	1	-	
Dependent	22	10	45.5	2.78	1.16–6.66	0.021
IADL						
Independent	274	59	21.5	1	-	
Dependent	130	39	30.0	1.56	0.97–2.51	0.065
Grip strength						
> 25th percentile	287	53	18.5	1	-	
≤ 25th percentile	117	45	38.5	2.76	1.71–4.45	< 0.001

ADL: Activities of daily living; IADL: Instrumental activities of daily living

* Wald Chi-square.

The adjustment of the variables of the distal level, in the hierarchical analysis, showed that being female and aged ≥ 65 years increase the chance of falls. At the intermediate level, poor sleep quality was the only statistically significant factor for a fall, regardless of the variables of that level and of the previous level. Only low muscle strength (≤ 25th percentile) was associated with a fall in the proximal level after adjusting for variables of the same level and of the previous levels (Table 4).

**Table 4:** Hierarchical logistic regression of the factors associated with a fall in persons aged 55 years or more. Cambé, State of Paraná, Southern Brazil, 2011 to 2012. (N = 372)

Variable	Total	Fall (yes)		Adjusted OR	95%CI	p^a^
	
	N	n	%			
Distal level^a^						
Gender						
Male	153	21	13.7	1	-	
Female	219	70	32.0	3.10	1.79–5.38	< 0.001
Age (years)						
55 to 64	191	33	17.3	1	-	
≥ 65	181	58	32.0	2.39	1.45–3.95	0.001
Intermediate level^b^						
Quality of sleep						
Good	218	42	19.3	1	-	
Poor	154	49	31.8	1.78	1.08–2.93	0.023
Proximal level^c^						
Muscle strength						
> 25th percentile	271	51	18.8	1	-	
≤ 25th percentile	101	40	39.6	2.31	1.34–3.97	0.003

^a^ Level adjusted for economic class, age, and gender.

^b^ Level adjusted for age, gender, sleep quality, depression, neoplasm, and body mass index.

^c^ Level adjusted for age, gender, sleep quality, depression, difficulty of going up/down the stairs, hearing impairment, activities of daily living, instrumental activities of daily living, and grip strength.

## DISCUSSION

The report of a fall in the population studied (24.3%) was lower when compared to studies carried out with an older population, although the associated factors have been similar. In a cohort of 1,415 individuals aged 65 years or more, living in the city of São Paulo, State of São Paulo, 30.9% reported fall on the year previous to the interview^16^. In other population-based studies in Brazil, the prevalence of falls over the past 12 months among the elderly aged 60 years or more was 27.6% in urban areas of one hundred municipalities of twenty-three States^22^, 27.1% in Montes Claros, State of Minas Gerais^17^, and 30.3% in a municipality in the State of Rio de Janeiro^15^. The lower frequency of falls identified in this research may be related to the composition of the sample, which includes adults aged from 55 years, and the predominance of individuals who are independent to carry out activities and instrumental activities of daily living.

This study has identified a significant association between falls and being female and of old age, regardless of economic class. Authors highlight the more frequent execution of domestic activities^16^ and greater loss of lean mass and muscle strength^14^ as possible causes of the higher incidence of falls among women. The advance of age leads to structural and functional changes that can reduce the ability to balance quickly and effectively, compromise the performance of motor skills, muscle strength, gait, and postural stability, and make an individual vulnerable to falls^1^.

Poor sleep quality has also been identified as a risk factor for falls even after adjusting for variables such as gender, age, and depression. This result was similar to that presented by Stone et al.^25^ in a study in the United States with 3,101 individuals, in which poor sleep quality assessed using the PSQI has been significantly associated with higher chance of falls.

After adjustment, the variable depression has not been associated with falls in this study, but depression can be related to a reduction in the speed of gait and loss of muscle strength, characteristics that are related to the occurrence of a fall^18^. According to Ricci et al.^19^, depressive symptoms deserve appropriate treatment and care, because of their debilitating consequences and association with falls, as they may have a devastating effect on the quality of life of the individual.

Low grip strength has also proved to be associated with falls, regardless of other variables such as sleep quality, depression, and difficulty going up/down the stairs. Visser et al.^27^ have identified higher risk of functional decline, falls, and mortality in older individuals with low muscle strength. The loss of muscle strength is considered an important indicator of frailty and is directly related with the increase in the number of falls, difficulty in going up or down the stairs, loss of agility, and fractures^3^.

Characteristics recognized as risk factors for falls, such as visual impairment, presence of other chronic diseases, and use of drugs^2^, have shown no association with this outcome in this research. Falls in the elderly are a multicausal event with numerous factors that act together and interact with each other^9^. This contributes to make it less likely that a given variable will stay associated with the event analyzed in the multiple model, in which other important factors were included.

This study has limitations. As this is a study developed in two moments, there were losses of individuals between the first and the second home interviews, although we have searched for information with neighbors and community health agents (ACS) and also from phone contacts, as strategies to minimize the losses. Another limitation is related to the fact that the fall was self-reported, a condition subject to the recall bias of the interviewee. Important characteristics for understanding the outcome, as environmental obstacles, wet surfaces, inadequate footwear^2^, among others, were not included in the analysis. Some health conditions, although included, did not make part of the multiple model, even though there was theoretical justification (such as the use of psychotropic drugs, hypertension, and diabetes), because of the low sample size. Some diseases, such as osteoarthritis, were not analyzed. The variable related to obesity grouped persons with low weight to normal weight or on the overweight range in the same category, which may have reduced the frequency of falls in this group and the difference in relation to persons with obesity. Furthermore, as the data was analyzed cross-sectionally, we cannot rule out the possibility of reverse causality, particularly in the relationship between falls and muscle strength. Falls can cause fractures – in a large study conducted in Brazil^22^, 11.0% of the elderly who suffered falls reported fractures – and, consequently, reduce muscle strength.

This research used data from a population-based study, which has allowed us to identify factors associated with falls in a population aged 55 years or more, with lower risk for this outcome than that observed in the elderly. These factors can be indicators of early loss of functional independence in this population. In addition, a hierarchical conceptual model was used for the study of factors associated with falls, and we have included demographic and socioeconomic characteristics related to lifestyle, presence of chronic diseases, and functional capacity.

The results found in this research are important to guide the actions of professionals who work in Family Health Units and Centers of Support to Family Health, as well as public policies, such as: encouragement of the practice of physical activity to strengthen muscles^6^, expanding of outdoor academies facilities, promotion of continuing education on environmental hazards and risk attitudes in the context of aging^12^, and identification of individuals at risk for falls, such as those with poor sleep quality, for proper care.

In summary, the frequency of falls identified in this research was lower than that of studies carried out only with the elderly population. In addition to being female and of old age, modifiable factors, such as poor sleep quality and low muscle strength, stood out as risk for falls. The identification of this profile may collaborate in directing health actions focused on the prevention of falls.

## Footnotes

a Instituto Brasileiro de Geografia e Estatística. Censo demográfico 2010: Paraná [cited 2011 May 10]. Available from: http://www.ibge.gov.br/home/estatistica/populacao/censo2010/tabelaspdff/total_populacao_parana.pdf

b Associação Brasileira de Empresas de Pesquisa. Critério de Classificação Econômica Brasil. São Paulo; 2009 [cited 2011 Oct 10]. Available from: http://www.abep.org/Servicos/Download.aspx?id=04

c World Health Organization. The anatomical therapeutic chemical classification system with defined daily doses (ATC/DDD). [cited 2012 Dec 05]. Available from: https://www.yumpu.com/en/document/view/4414280/guidelines-for-atc-classification-and-ddd-assignment-whocc
